# Synthesis and Evaluation of ^125^I-IMPY: Application in Neuroblastoma Tracing and Treatment

**DOI:** 10.3390/life15060930

**Published:** 2025-06-09

**Authors:** Tsung-Ming Wang, Shih-Chang Chuang, Kuo-Chen Hung, Chang-Ching Yu, Tzu-Chuan Ho, Kuo-Pin Chuang, Cheng-Hui Yuan, Ming-Hui Yang, Yu-Chang Tyan

**Affiliations:** 1 Department of Medical Imaging, China Medical University Hospital, Taichung 404, Taiwan; 2 Division of General and Digestive Surgery, Department of Surgery, Kaohsiung Medical University Hospital, Kaohsiung Medical University, Kaohsiung 807, Taiwan; 3 School of Medicine, College of Medicine, Kaohsiung Medical University, Kaohsiung 807, Taiwan; 4 Department of Nuclear Medicine, Kaohsiung Veterans General Hospital, Kaohsiung 813, Taiwan; 5 Department of Medical Imaging and Radiological Sciences, Kaohsiung Medical University, Kaohsiung 807, Taiwan; 6 Graduate Institute of Animal Vaccine Technology, College of Veterinary Medicine, National Pingtung University of Science and Technology, Pingtung 912, Taiwan; 7 Mass Spectrometry Laboratory, Department of Chemistry, National University of Singapore, Singapore 119077, Singapore; 8 Department of Medical Research, Kaohsiung Medical University Hospital, Kaohsiung 807, Taiwan; 9 Center for Tropical Medicine and Infectious Disease Research, Kaohsiung Medical University, Kaohsiung 807, Taiwan; 10 Center for Cancer Research, Kaohsiung Medical University, Kaohsiung 807, Taiwan

**Keywords:** isotope labeling, IMPY, radioactive iodine, iodogen, neuroblastoma

## Abstract

Background: Radiolabeled compounds can serve as diagnostic or therapeutic agents depending on the characteristics of the isotopes used. IMPY (6-iodo-2-(4′-dimethylamino)-phenyl-imidazo[1,2-a]pyridine) is a lipophilic derivative of thioflavin-T, designed to function as a tracer when labeled with radioactive iodine. While it has been primarily studied for imaging applications, its potential therapeutic effects when labeled with iodine-125 (^125^I) remain to be explored. Methods: In this study, IMPY was synthesized and labeled with ^125^I for therapeutic purposes. Three different labeling methods were employed: isotope exchange reaction, redox reaction, and the Iodogen technique. The radiochemical yield of each method was determined to identify the most effective approach. Additionally, the effects of ^125^I-IMPY on neuroblastoma cells were evaluated by assessing its toxicity and cellular uptake. Results: The radiochemical yields for the isotope exchange reaction, redox reaction, and Iodogen technique were found to be 0.96%, 10.74%, and 96.52%, respectively. The Iodogen technique exhibited the highest yield, exceeding 90% even after 48 h, making it the most efficient method. Furthermore, the impact of ^125^I-IMPY on neuroblastoma cells was analyzed, revealing significant cellular uptake and potential therapeutic effects. Conclusions: This study demonstrated that the Iodogen technique is the most effective method for labeling IMPY with ^125^I. The high labeling efficiency and observed cellular effects suggest that ^125^I-IMPY could be considered not only as a tracer but also as a potential therapeutic agent for neuroblastoma. Further studies are needed to explore its full therapeutic potential and mechanism of action.

## 1. Introduction

Neuroblastoma is a type of cancer that arises from immature nerve cells, primarily affecting young children. It most commonly develops in the adrenal glands, located above the kidneys, but can also occur in nerve tissues along the spine, chest, abdomen, or pelvis. Neuroblastoma is the most common extracranial solid tumor in children and is known for its heterogeneous behavior, ranging from spontaneous regression to aggressive progression [1]. The disease can be associated with genetic mutations, such as MYCN oncogene amplification, which is linked to poor prognosis [2,3].

SH-SY5Y cells, a cloned subline of a neuroblastoma cell line derived from a metastatic bone tumor, are commonly used as an in vitro model for neuronal function and cell differentiation. They have been widely applied in neuroscience research, including studies on Parkinson’s disease [4], Alzheimer’s disease, neurotoxicity, ischemia, and amyotrophic lateral sclerosis (ALS) [5,6,7]. Additionally, they serve as a valuable tool for investigating other characteristics of brain cells and neurogenesis [8].

Radiolabeled compounds are useful as both diagnostic and therapeutic agents. In diagnostics, they play a crucial role in identifying subtle indicators of oncological and neurological disorders [9]. In treatment, radiopharmaceuticals can be designed with various carriers to damage or eliminate tumor cells [10]. Some molecules function as both diagnostic and therapeutic agents, a concept known as theranostics [11]. Similarly, certain elements can exhibit a dual role, as exemplified by radioactive iodine-containing compounds, which can function as either imaging or therapeutic agents, depending on their decay mode, and whose versatility is enabled by radiohalogenation. There are over 30 isotopes of iodine (I), among which Iodine-127 is a stable, non-radioactive isotope. Four of these radionuclides have been adapted for medical use: Iodine-123 and Iodine-124 produce high-quality images, while Iodine-125 and Iodine-131 can be used for therapeutic purposes. Most iodine isotopes are not naturally occurring; for instance, Iodine-131 is produced from the decay of Tellurium-131 through beta emission [12]. Furthermore, because compounds must be labeled to function as radiopharmaceuticals, radioiodination is a crucial procedure. An effective radioiodination process adds radioactive iodine to a compound or biomolecule without altering its physicochemical or biological properties [13].

Imidazoles are organic, heterocyclic compounds containing two non-adjacent nitrogen atoms (meta-substituted). Their structural versatility enables wide-ranging pharmaceutical and materials science applications. For instance, benzimidazole derivatives, featuring a benzene ring fused to an imidazole, have shown effectiveness as fungicides, anthelmintics, and agents for antiviral, antitumor, and antihypertensive therapies [14]. Zeolitic imidazolate frameworks (ZIFs), when combined with bioactive molecules such as amino acids, peptides, proteins, or nucleotides, have also been employed in biosensor development [15]. One notable imidazole derivative is 6-Iodo-2-(4′-dimethylamino)-phenyl-imidazo[1,2-a]pyridine (IMPY), a heterocyclic compound derived from the thioflavin-T structure. It features a dimethylamino-substituted phenyl group at the 2-position and an iodine atom at the 6-position of the imidazo[1,2-a]pyridine core. The unique structural and chemical characteristics of IMPY make it a valuable scaffold for drug discovery, with potential as a lead compound targeting biological receptors or enzymes such as GABA-A receptors and kinases [16].

IMPY has been extensively studied as a radiolabeled probe for imaging β-amyloid plaques in Alzheimer’s disease [17]. The iodine moiety allows for radiolabeling with isotopes such as iodine-123, iodine-125, or iodine-131, facilitating its use in diagnostic imaging [18]. For example, ^18^F-labeled styryltriazole and imidazo[1,2-a]pyridine derivatives [19], as well as ^123^I-labeled IMPY, have been employed to selectively image β-amyloid plaques [20], while ^125^I-labeled variants have shown strong binding affinity to post-mortem Alzheimer’s disease brain tissue, indicating their potential for detecting Tau tangles [21]. Notably, IMPY derivatives also exhibit good blood–brain barrier permeability, further underscoring their relevance in neuroimaging research. Beyond neurodegenerative applications, IMPY has shown additional therapeutic potential, including antiviral [22] and anti-angiogenic [23] properties.

In this study, a novel imidazole-based compound was synthesized through the regioselective formation of three heterocyclic bonds with targeted substitutions at the N-1, C-2, and C-4 positions [24]. To expand its potential applications, we radiolabeled the compound with ^125^I, a radionuclide known for its favorable nuclear properties, including a 60-day half-life and gamma emissions ideal for targeted radiotherapy with minimized damage to surrounding healthy tissues. We compared different radiolabeling methods to determine optimal labeling efficiency, and their effects on neuroblastoma cells were evaluated.

## 2. Materials and Methods

### 2.1. Compound Syntheses

All reagents were purchased from commercial suppliers and used without further purification unless stated otherwise. Column chromatography and thin-layer chromatography (TLC) were performed on silica gel 60 F_254_ (230–400 mesh, Merck, Darmstadt, Germany) and silica gel 60 glass plates (Merck, Darmstadt, Germany), respectively. Preparative thin-layer chromatography (PTLC) was conducted on silica gel plates with a fluorescent indicator, visualized under 254 nm UV light.

An ethanol (J.T.Baker #8006-05, Landsmeer, The Netherlands) solution containing 5-iodo-2-pyridinamine **1** (100 mg) (IPA, Matrix #001779, Columbia, SC, USA) and 4-(dimethylamino)phenacyl bromide **2** (120 mg) (DMPB, Apollo Scientific 102 #OR3530, Manchester, UK) was refluxed for 2 h, during which time samples were taken at hourly intervals for analysis by TLC (Macherey-Nagel #818133, Rotherham, UK). After cooling, NaHCO_3_ (Sigma #S5761, St. Louis, MO, USA) was added to the solution, which was then refluxed for another hour. Subsequently, the reaction mixture was absorbed onto silica (0.04–0.063 mm, Macherey-Nagel #815381, Rotherham, UK). The crude product was purified by column chromatography using a solvent mixture of ethyl acetate and n-hexane (1:6) (Ethyl acetate, Sigma #270989: n-Hexane, Sigma #296090, St. Louis, MO, USA) to yield 6-iodo-2-(4′-dimethylamino)-phenyl-imidazo[1,2-a]pyridine **3** (86.1 mg, 47.4%). ^1^H NMR (200 MHz, CDCl_3_): δ 2.998 (s, 6H), 6.633 (d, 2H), 7.265 (dd, 1H), 7.381 (d, 1H), 7.643 (s, 1H), 7.788 (d, 2H), 8.302 (s, 1H). ^13^C NMR (200 MHz, CDCl_3_): δ 40.415, 98.274, 106.225, 112.401, 117.970, 121.202, 127.060, 130.094, 131.915, 144.085, 146.953, 150.580.

A mixture of 6-iodo-2-(4′-dimethylamino)-phenyl-imidazo[1,2-a]pyridine **3** (200 mg) and Pd(Ph_3_P)_4_ (Sigma #216666, St. Louis, MO, USA) in a solution of 1,4-dioxane (Merk #1115097, St. Louis, MO, USA), triethylamine (Sigma #T0886, St. Louis, MO, USA), and (Bu_3_Sn)_2_ **4** (80 mg) (Alfa Aesar #A12007, Waltham, MA USA) was stirred at 90 °C for 1 h. The solvent was then removed, and the residue was purified by PTLC (ethyl acetate: n-hexane = 1:1 as the developing solvent) to yield SnMPY **5** (52.9 mg, 18.3%). ^1^H NMR (200 MHz, CDCl_3_): δ 0.901 (t, 9H), 1.106 (t, 6H), 1.330 (m, 6H), 1.567 (m, 6H), 2.994 (s, 6H), 6.791 (d, 2H), 7.111 (d, 1H), 7.576 (d, 1H), 7.710 (s, 1H), 7.848 (d, 2H), 7.954 (s, 1H). ^13^C NMR (200 MHz, CDCl_3_): δ 9.748, 13.617, 27.274, 28.974, 40.476, 105.573, 112.477, 116.605, 121.369, 122.204, 126.953, 129.836, 130.747, 145.511, 145.693, 150.322.

**Scheme 1:**
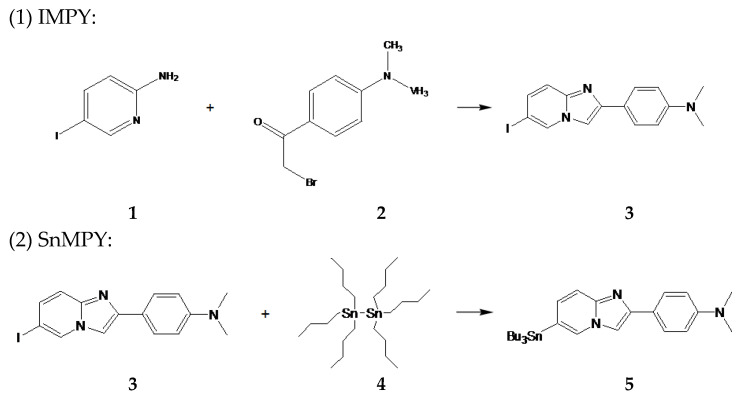
Chemical scheme depicting the preparation route for IMPY and SnMPY.

The chemical synthesis reactions and reaction equations of IMPY and SnMPY are shown below (Scheme 1):

### 2.2. Structural Analysis of Compounds

The ^1^H NMR and ^13^C NMR spectra of the synthesized compounds were recorded on NMR spectrometers (Varian NMR spectrometer Gemini 2000, Palo Alto, CA, USA), with chemical shifts reported in ppm (δ values) and referenced to CDCl_3_. Mass spectra were obtained using liquid chromatography coupled with mass spectrometry (LC-MS), specifically a Waters 2695 Separations Module with a Waters Micromass ZQ (Milford, MA, USA).

### 2.3. Radiolabeling

IMPY was labeled with ^125^I ion using three methods: isotopic exchange, oxidative iodination, and the Iodogen method.

For the isotopic exchange method, IMPY was incubated in a Na^125^I (I-RB-4, Institute of Isotopes) solution for 2 h, allowing the ^125^I ion in the solution to exchange with labile iodine in IMPY. After incubation, the mixture was purified using a C18 SPE cartridge (Thermo #60108-302, Waltham, MA, USA) to separate unlabeled ^125^I ions and IMPY from ^125^I-IMPY.

For oxidative iodination, 50 μL of 100% ethanol was added to 200 μg of SnMPY, then thoroughly mixed with 300 μL of Na^125^I (activity approximately 1 mCi). Next, 100 μL of 5% H_2_O_2_ solution (prepared by mixing 58.3 μL of water, 25 μL of glacial acetic acid, and 16.7 μL of 30% H_2_O_2_) was added, and the mixture was shaken for 15 min. After the reaction, 300 μL of 39% NaHSO_3_ (Sigma #243973, St. Louis, MO, USA) was added to stop the iodination reaction, followed by the immediate addition of 2 mL of saturated KH_2_PO_4_ (Riedel-de Haën #S240697, Seelze, Germany). The resulting solution was passed through a C18 SPE cartridge, washed with 9 mL of water, and then eluted with 2 mL of 40% ethanol to remove impurities. Finally, the desired product, ^125^I-IMPY, was eluted using 2 mL of 60% ethanol.

For the Iodogen method, 300 μg of SnMPY and 10 μL of phosphate buffer (pH 8.0) were added to a coating tube containing 2 mg of Iodogen (Thermo #28600, Waltham, MA, USA) and mixed. Then, approximately 200 μCi of Na^125^I was added and mixed. The reaction was allowed to proceed at room temperature for 20 min and then the reaction solution was removed. To terminate the reaction and remove unbound ^125^I, the Iodogen tube was rinsed with 1 mL of deionized water. The radiolabeled IMPY was then eluted by rinsing the tube with 1 mL of ethanol. A TLC reader (BrightSpec bSCAN-V3, Niel, Belgium) was used to determine the radiochemical conversion and Rf values.

For the method of radiochemical yield (RCY) determination, the dose of iodinated IMPY (DL) is divided by the sum of the dose of iodinated IMPY (DL) and the dose of free ^125^I ion (DF). The RCY was calculated using the following formula: $RCY=\frac{D_{L}}{D_{L}+D_{F}}$.

**Scheme 2:**
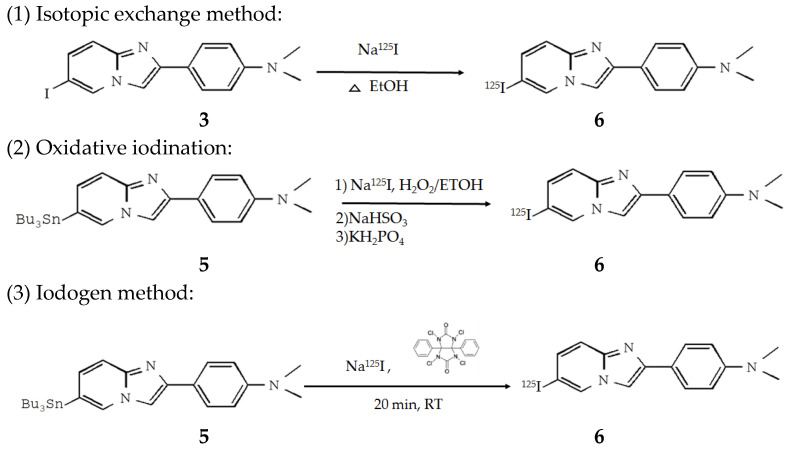
Chemical reactions of iodine-125 labeling.

The radiolabeling procedures to obtain ^125^I-IMPY are shown below (Scheme 2):

### 2.4. Radiolabeling Stability Test

After radiolabeling the compound, TLC was used to measure the ratio of iodinated IMPY to free iodine in order to assess the stability of the compound. Stability tests were conducted at 0, 24, and 48 h after preparation, monitoring the ratio of iodinated IMPY to free ^125^I and calculating its change over time.

### 2.5. Cell Culture

A neuroblastoma cell line (SH-SY5Y) was cultured in Dulbecco’s modified Eagle’s medium (DMEM) supplemented with 10% fetal bovine serum (FBS), L-glutamine (2 mM), streptomycin (100 μg/mL), and penicillin (100 units/mL). The cell line was maintained at 37 °C in a humidified CO_2_ incubator.

### 2.6. Cell Viability and Cellular Uptake

3-(4,5-Dimethylthiazol-2-yl)-2,5-diphenyltetrazolium bromide (MTT) is a yellow compound that can be catalyzed by succinate dehydrogenase (SDH) in the mitochondria of viable cells, generating purple formazan crystals. By dissolving these formazan crystals in dimethyl sulfoxide (DMSO), their absorbance can be used to evaluate cell viability, making MTT assays a common method for assessing the effects of drugs on cell survival.

To evaluate the effect of ^125^I-IMPY on SH-SY5Y cell viability, SH-SY5Y cells (2 × 10^4^ cells per well, 200 μL per well) were seeded into a 96-well plate. After 24 h, the culture medium was replaced, and the cells were incubated with ^125^I-IMPY at four different doses (5, 10, 20, and 100 μCi) for 24 h. During cell culture, lead plates were used as shielding barriers to separate cells receiving different radiation doses. Following incubation, the medium was removed, and 100 μL of fresh culture medium was added to each well, followed by the addition of 10 μL of 12 mM MTT solution. The plate was incubated at 37 °C in a 5% CO_2_ incubator for 4 h.

After incubation, 85 μL of the MTT-containing medium was removed, leaving a residual volume of 25 μL. Then, 100 μL of DMSO was added to each well, mixed thoroughly, and incubated at 37 °C in a 5% CO_2_ incubator for 10 min. The absorbance at 540 nm was then measured using a microplate reader (BioTek Synergy H1, Agilent, Santa Clara, CA, USA).

To determine the cellular uptake, cells (1.5 × 10^5^) were seeded overnight in 10 cm dishes and incubated with 2 μCi of ^125^I-IMPY. After 24 and 48 h of incubation at 37 °C with 5% CO_2_, the cells were collected, and the isotopic activity was analyzed using a well-type scintillation counter (Scalar Ratemeter SR-7, Nuclear Enterprises Ltd., Edinburgh, UK).

### 2.7. LDH Assay

The cell cytotoxicity study was conducted using the lactate dehydrogenase (LDH) leakage assay, which measures LDH released into the culture medium. The assay is based on the conversion of lactate to pyruvate in the presence of LDH, accompanied by the reduction of NAD. The resulting formation of NADH led to a measurable change in absorbance at 340 nm using a microplate reader (BioTek Synergy H1, Agilent, Santa Clara, CA, USA).

### 2.8. Immunofluorescence Staining

After treating with various concentrations of ^1^^25^I-IMPY, cells were fixed with cold methanol. The nuclei and cytoskeleton of the cells were stained with 4′-6-diamidino-2-phenylindole (DAPI, Thermo# 62247, Waltham, MA, USA) and vimentin (EPITOMICS #2862-1, Waltham, MA, USA), respectively. In addition, an Apoptosis Detection Kit (ApopTag#S7165, Merk, St. Louis, MO, USA) was used according to the manufacture’ protocol. The DNA break was detected and stained with a rhodamine fluorochrome (FLoid Cell Fluorescence Imaging Station, Invitrogen, Waltham, MA, USA).

### 2.9. Western Blot

Protein extracts were prepared in RIPA cell lysis buffer. Each protein sample (1 mg/mL, 10 μL) was electrophoresed on a precast gel (NuPAGE^®^ Novex^®^ 4–12% Bis-Tris Gel, 1.5 mm, 10 wells; Invitrogen™, Waltham, MA, USA). Proteins were then transferred from the gel to a polyvinylidene difluoride (PVDF) membrane using a semidry transfer system (Criterion Blotter, Bio-Rad, Hercules, CA, USA) at 100 V for 60 min and subsequently blocked with 5% milk in PBS (pH 7.4) containing 0.05% Tween-20. The membranes were incubated overnight with the primary antibody (γ-H2AX, SAB5600038, Sigma-Aldrich, St. Louis, MO, USA, 1:500 dilution or β-actin, AC-15, Sigma-Aldrich, St. Louis, MO, USA, 1:5000 dilution).

Following rinsing with PBST, the membranes were incubated with HRP-conjugated secondary antibodies (1:10,000 dilution) for 1 h. Protein detection was performed using an enhanced chemiluminescence (ECL) system, and quantitative analysis was carried out using the ImageQuant-TL-7.0 software (GE Healthcare, Amsterdam, The Netherlands).

### 2.10. Statistical Analysis

*p*-Values were calculated using the unpaired two-tailed Student’s *t*-test with MS excel and GraphPad Prism 5.0 (GraphPad, La Jolla, CA, USA). Data were considered statistically significant at *p* < 0.05.

## 3. Results

### 3.1. Compound Synthesis

After synthesizing IMPY and SnMPY, compounds were characterized using liquid chromatography–mass spectrometry, and ^1^H- and ^13^C- nuclear magnetic resonance spectroscopy. As shown in Supplementary Figure S1, a compound with *m*/*z* = 364 was eluted at 27 min, which corresponded with the molecular weight of IMPY, 363. For SnMPY, the compound was eluted at 44 min with a peak at *m*/*z* = 528, which agreed with that of SnMPY.

From the ^1^H NMR and ^13^C NMR spectra (Supplementary Figures S2 and S3), the observed number of hydrogen and carbon atoms matched that of IMPY and SnMPY, and the chemical shifts were consistent with a previous study [25], confirming the correctness of the compound. After IMPY and SnMPY were synthesized and their structures confirmed, the radiolabeling proceeded.

### 3.2. Radiolabeling of IMPY with Isotope ^125^I

**Figure 1:**
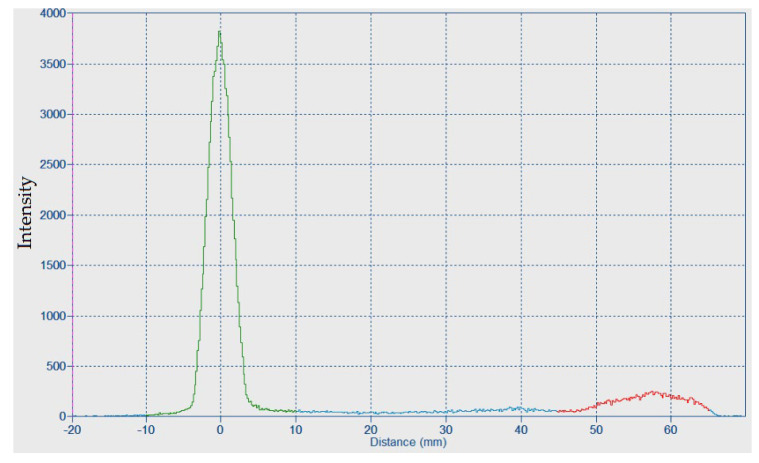
Radiochromatograms of ^125^I-IMPY using PBS as the developing solvent, with a development distance of 7 cm.

After characterizing compounds of IMPY and SnMPY, the isotopic labeling proceeded using three methods—isotopic exchange, oxidative iodination, and the Iodogen method. The radiochemical yield using the isotopic exchange method was very low. Thus, oxidative iodination and the Iodogen method were adapted. After the purification process, the purities of ^125^I-IMPY were detected using TLC and showed only a single peak for the product of the Iodogen method (Figure 1).

Table 1 shows the radiochemical yield of ^125^I-IMPY among the methods of isotopic exchange, oxidative iodination, and Iodogen iodination. The isotopic exchange method had the lowest radiochemical yield, 0.96%, and the Iodogen iodination had the highest radiochemical yield, 96.52%.

In the isotope exchange method, ^125^I-IMPY was labeled and then separated from unbound ^125^I ions using a C18 SPE cartridge. The final product was eluted with 100% ethanol, the highest polarity solvent, and the radioactivity was measured to calculate the radiochemical yield. The calculated yield showed that only 0.96% of IMPY was successfully labeled with the ^125^I ion. Compared to other labeling methods, this method resulted in a very low radiochemical yield, making it unsuitable for producing sufficient ^125^I-IMPY.

In the oxidative iodination method, ^125^I-IMPY appeared in fractions eluted with 60% and 100% ethanol through the C18 SPE cartridge. The fraction eluted with 60% ethanol contained fewer impurities and less free ^125^I ions. By concentrating this fraction using a lyophilizer, compared with the isotope exchange method, a higher concentration of ^125^I-IMPY was obtained. The radiochemical yield was measured as 10.74%. According to a previous study, Kung et al. employed high-performance liquid chromatography (HPLC) for purification, achieving higher radiochemical yields ranging from 30% to 45% [17]. HPLC offers superior resolution and efficiency in separating ^125^I-IMPY from impurities and free ^125^I ions compared to SPE. This difference in purification technique could significantly impact the overall yield and purity of the final product. Additionally, the presence of impurities generated during the synthesis process can further reduce the radiochemical yield. Impurities may interfere with the labeling efficiency and complicate the purification process, leading to lower overall yields. On the other hand, the Iodogen method employs mild oxidizing conditions that enable efficient iodination while minimizing oxidative damage to sensitive biomolecules. Consequently, we selected this method for our radioiodination procedures.

In the Iodogen iodination method, a pre-coated Iodogen tube was used to label ^125^I onto IMPY. PBS (1×) was used as the developing solvent, with chromatography starting at 0 cm and ending at 7 cm on the TLC plate. Due to the significant molecular weight difference between labeled and free iodine, the ^125^I-IMPY, having a larger molecular weight, remained near the origin, whereas the free ^125^I ion moved toward the endpoint with PBS. The proportion of free iodine to iodine bound to IMPY can be determined by analyzing the peak areas obtained from TLC. The final radiochemical conversion for ^125^I-IMPY was 96.52 ± 0.39%.

**Figure 2:**
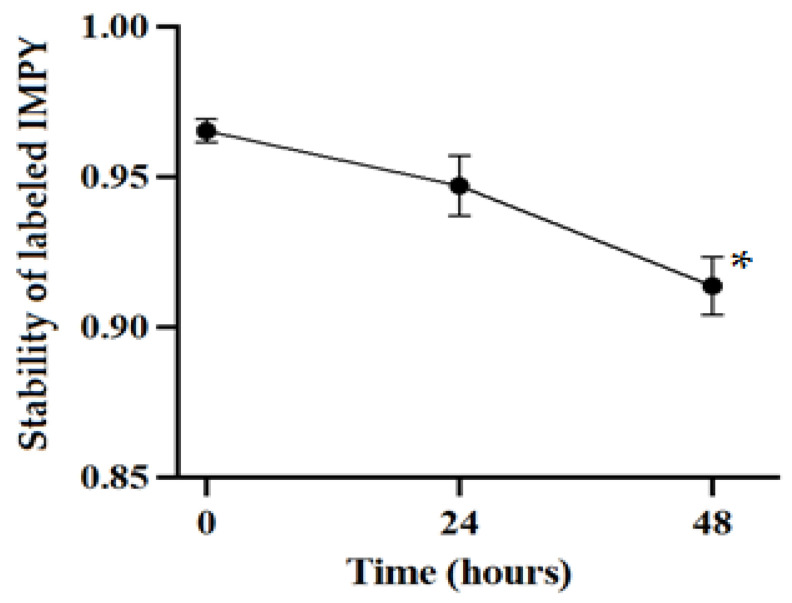
Stability analysis of IMPY labeled with ^125^I after 24 and 48 h. Results are means ± the standard deviation (* *p* < 0.05, *N* = 6).

Among the three labeling methods, the Iodogen iodination method achieved the highest radiochemical yield. Stability tests were performed using the bSCAN TLC Reader at 0, 24, and 48 h post-labeling. As shown in Figure 2, the radiochemical purity of ^125^I-IMPY at 48 h post-labeling remained at 93.38 ± 3.54%, demonstrating that over 90% of the ^125^I ion remained bound to the drug, even after 48 h.

### 3.3. In Vitro Study

**Figure 3:**
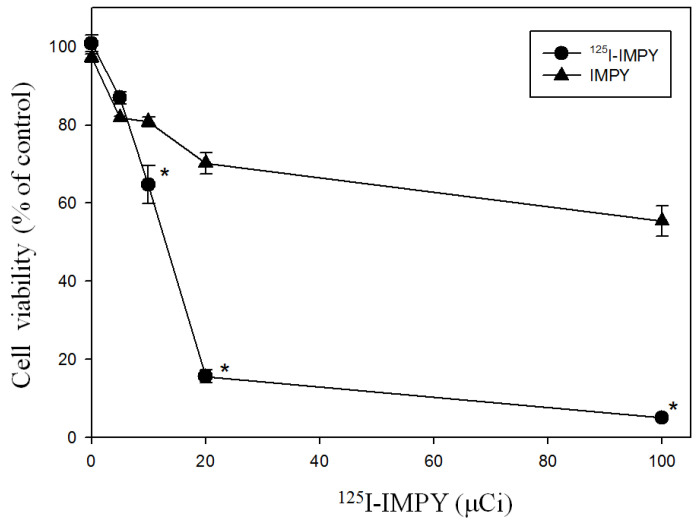
SH-SY5Y cells were treated with various doses of ^125^I-IMPY and IMPY. The percentages of viable cells were normalized to the solvent-treated control. Results are means ± the standard deviation (* *p* < 0.05, *N* = 6).

Since IMPY was originally synthesized from SnMPY and developed for brain imaging, neuroblastoma cells (SH-SY5Y) were used in a viability assay with IMPY. As shown in Figure 3, cell viability decreased with increasing doses. Compared to an equivalent amount of IMPY without the radioactive iodine label, an increased radiation dose significantly decreased cell viability.

**Figure 4:**
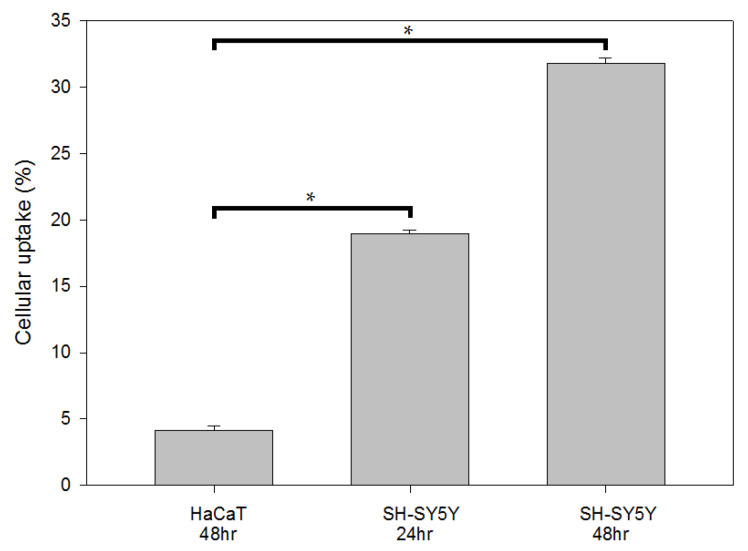
The cellular uptake of ^125^I-IMPY for HaCaT and SH-SY5Y cells. Results are means ± the standard deviation (* *p* < 0.05, *N* = 6).

Cellular uptake is a crucial biological process that allows cells to protect themselves from foreign materials by enhancing their cellular uptake efficiency. The effects of ^125^I-IMPY on neuroblastoma cells (SH-SY5Y) were evaluated by measuring their cellular uptake. To quantify cellular uptake, radiation counts of ^125^I-IMPY that went through cellular uptake by SH-SY5Y cells were measured using a well-type scintillation counter. As shown in Figure 4, the uptake rate was approximately 18.96% at 24 h, increasing to 32.07% at 48 h, indicating that longer incubation enhances cellular uptake. The results demonstrated a significant increase in ^125^I-IMPY uptake between the first and second days. Given that the absorbed dose in SH-SY5Y cells was notably higher than in normal HaCaT cells, ^125^I-IMPY appears to be a suitable tracer for neuroblastoma. Additionally, apoptosis levels increased with higher ^125^I-IMPY concentrations, further supporting its potential applications.

At low doses, ^125^I-IMPY exhibited minimal cytotoxic effects on SH-SY5Y cells. When cell viability was maintained at 90%, the tolerable concentration of the drug was approximately 4.35 μM. The IC_50_ value, representing the concentration at which 50% of the cells were inhibited, was 12.17 μM. These two values differed significantly (*p* < 0.05).

**Figure 5:**
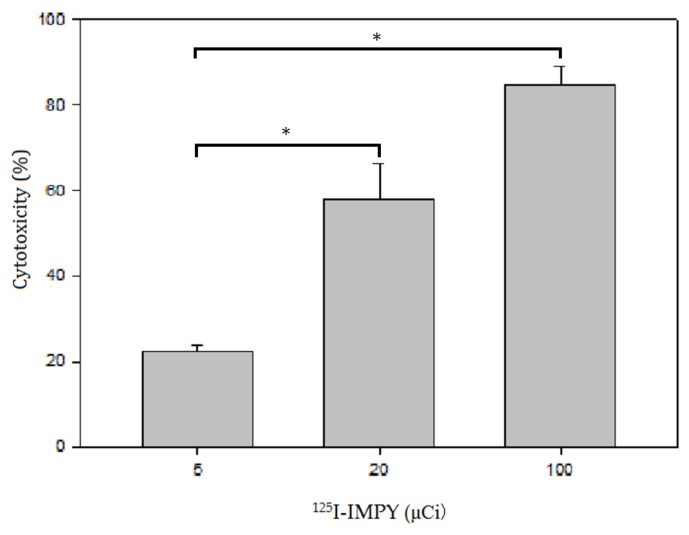
LDH cytotoxicity assay. Percentage of cytotoxic activity, as measured by LDH released from SH-SY5Y cells treated with 5, 20, or 100 μCi ^125^I-IMPY for 24 h. The results represent the means of three independent determinations. (* *p* < 0.05, *N* = 6).

To study the effect of radiation on SH-SY5Y cells, the LDH assay was used to assess cell viability under different radiation doses of ^125^I-IMPY. Figure 5 shows that radiation doses higher than 20 μCi were toxic to cells after 24 h of exposure.

This result may be attributed to the fact that SH-SY5Y cells are neuronal cells, and IMPY was originally designed as a brain imaging agent, specifically targeting regions with a high density of neuronal cells. As a result, its cytotoxic effects on neuronal cells are relatively low.

### 3.4. Cellular Fluorescent Immunostaining and Apoptosis

Apoptosis was assessed using fluorescent immunostaining. Three different staining agents were used in this experiment: Apoptosis Detection Kit to observe apoptosis; vimentin to stain the cytoplasm; and DAPI to stain the nucleus.

**Figure 6:**
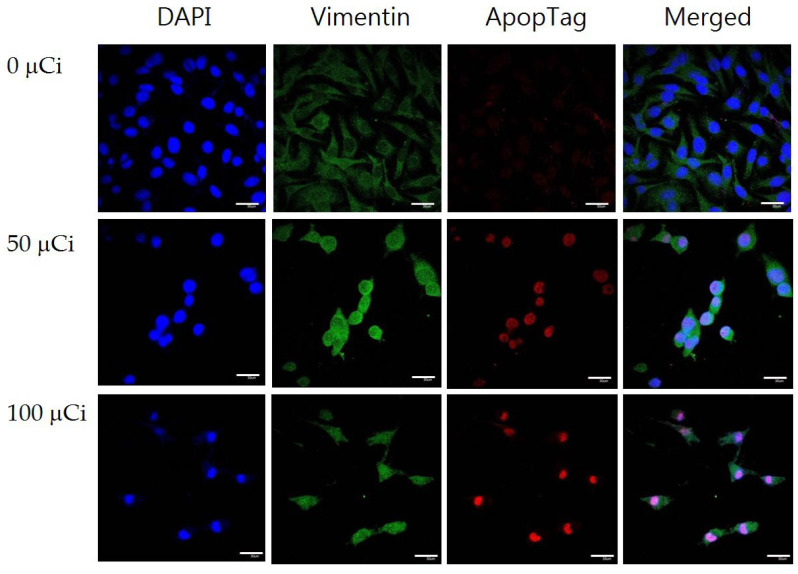
Images for SH-SY5Y cells treated with 0, 50, or 100 μM of ^125^I-IMPY. The nuclei and cytoskeleton of the cells were stained with DAPI (blue) and vimentin (green), respectively. ApopTag (an apoptotic marker) is shown in red. Scale bar represents 30 μm.

SH-SY5Y cells are characterized by their elongated structures on both sides. As shown in the fluorescent staining results (Figure 6), as the drug concentration increased, the elongated structures gradually disappeared, the number of cells decreased, and the extent of apoptosis progressively increased.

**Figure 7:**
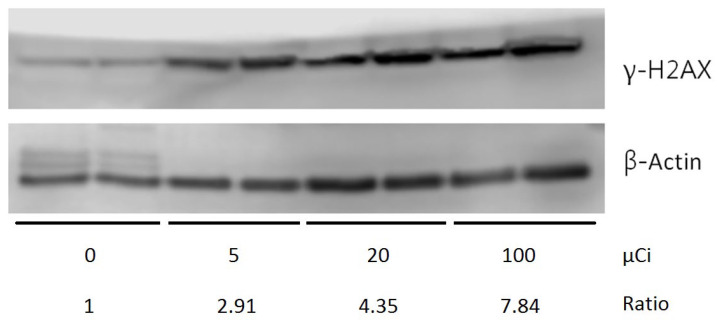
Western blot and quantitative densitometry analysis of γ-H2AX and β-actin protein expression in SH-SY5Y cells treated with 0, 5, 20, or 100 μCi ^125^I-IMPY for 24 h. β-actin was used to normalize the protein expression.

After exposure to ionizing radiation that causes DNA double-strand breaks, histone H2AX was rapidly phosphorylated, forming γ-H2AX nuclear foci. The early-activated protein kinase ATM played a critical role in this phosphorylation process. At the cellular level, γ-H2AX quantitatively reflected radiation-induced DNA damage, supporting its widespread use as a biomarker for dose-dependent ionizing radiation effects. Figure 7 shows the expression levels of γ-H2AX in cells treated with radiolabeled ^125^I-IMPY. As the dose increased, γ-H2AX expression also increased, indicating that the cells experienced more severe radiation damage.

## 4. Discussion

As a promising neuroimaging agent in nuclear medicine, ^125^I-IMPY exhibits high specificity, favorable pharmacokinetics with rapid metabolism, and low uptake by surrounding normal cells. This study aimed to synthesize IMPY, label it with ^125^I ion using various methods, and evaluate its cytotoxic effects while monitoring cellular changes through fluorescent immunostaining. Three different methods were employed for labeling ^125^I-IMPY: isotope exchange labeling, oxidative iodination, and Iodogen iodination. The isotope exchange method replaces a non-radioactive iodine atom on an aromatic ring with a radioactive one, while oxidative iodination utilizes radioiododestannylation—both proceeding via electrophilic substitution reactions. In contrast, the Iodogen method follows a nucleophilic substitution reaction [26]. Although radioiododestannylation is widely used in laboratory settings [27], its labeling efficiency was significantly lower compared to the Iodogen method, which achieved a radiochemical yield of 96.52%. Even after 48 h, the labeling rate remained at 93.38%, demonstrating excellent stability. Notably, we are the first group to successfully label ^125^I into IMPY using the Iodogen method, highlighting its superior efficiency. These findings suggest that Iodogen iodination is the most effective method for ^125^I-IMPY labeling and warrants further exploration for potential applications.

Initially, ^123^I-IMPY was developed as a SPECT imaging agent for β-amyloid labeling; however, during preclinical trials, its signal-to-noise ratio proved unsatisfactory [28]. Consequently, the continued development of novel β-amyloid probes led to the removal of ^123^I-IMPY from the list of potential imaging agents [28]. Among the available isotopes, ^125^I is cost-effective, widely available, and associated with relatively few complications [26]. Therefore, we labeled ^125^I into IMPY as a potential therapeutic agent for neuroendocrine tumors and evaluated its toxicity in neuroblastoma cells. To assess the effect of ^125^I-IMPY on SH-SY5Y cell growth, we performed a serial dilution analysis across a range of concentrations. Although ^125^I-IMPY at lower doses inhibited cell growth, its cytotoxicity remained within the microcurie (µCi) range, indicating relatively low toxicity to these cells. When comparing the ^125^I-IMPY cell growth inhibition curve, we observed that at doses between 15 and 100 µM, cell viability sharply declined to approximately 60%.

The radiochemical yield results indicate that the Iodogen iodination method achieved the highest yield. In cellular experiments, SH-SY5Y cells exhibited significantly higher uptake rates compared to normal HaCaT cells, with uptake on the second day being markedly higher than that on the first day. In the cytotoxicity assay, SH-SY5Y cells maintained 90% viability at a dose that was approximately half of the IC_50_ value. This may be attributed to the fact that SH-SY5Y cells are neuronal in origin, while ^125^I-IMPY is a brain imaging agent designed for regions rich in neuronal cells. Consequently, SH-SY5Y cells were more sensitive to ^125^I-IMPY, reaching the IC_50_ at double the tolerable dose. Furthermore, fluorescent immunostaining demonstrated that as the drug concentration increased, SH-SY5Y cell morphology changed, cell numbers decreased, and apoptosis increased.

The phosphorylated form of the histone protein H2AX, known as γ-H2AX, is a well-established biomarker for DNA double-strand breaks (DSBs). Upon the occurrence of DSBs, such as those induced by ionizing radiation or DNA-damaging agents, H2AX is rapidly phosphorylated at the site of damage, leading to the formation of γ-H2AX foci. These foci serve as platforms for the recruitment and localization of DNA repair proteins, thereby playing a critical role in the DNA damage response and repair mechanisms [29,30]. Quantifying γ-H2AX levels is widely used to evaluate both the extent of DNA damage and the repair efficiency of cells.

In the context of our study, radiolabeled IMPY may also exert radiopharmaceutical effects, including the induction of DNA damage in tumor cells. This suggests a dual role for radiolabeled IMPY as a tumor-targeting tracer and as a therapeutic agent capable of eliciting cellular responses such as γ-H2AX formation, which is indicative of DNA damage. Further investigation is warranted to explore this potential and to better understand the therapeutic implications of radiolabeled IMPY in cancer treatment.

## 5. Conclusions

This study provided valuable insights into the cytotoxicity and tolerance of SH-SY5Y cells to ^125^I-IMPY. Our findings suggest that radiolabeled IMPY could be further explored for applications in molecular imaging and radiotherapy, particularly in the context of neurodegenerative diseases or tumor targeting. Future studies will focus on in vivo evaluations to further validate the compound’s potential. These investigations will include assessments of biodistribution, pharmacokinetics, and target specificity in appropriate animal models. These perspectives will guide the translational development of radiolabeled IMPY as a multifunctional probe in nuclear medicine.

## Figures and Tables

**Table 1:** The radiochemical yield of ^125^I-IMPY among the methods of isotopic exchange, oxidative iodination, and Iodogen iodination.

Labeling Method	Radiochemical Yield (%)	SD (%)
Isotopic exchange	0.96	0.038
Oxidative iodination	10.74	0.343
Iodogen iodination	96.52	0.390

## Data Availability

The raw data supporting the conclusions of this article will be made available from the corresponding authors upon reasonable request.

## Supplementary Materials

The following supporting information can be downloaded at: https://www.mdpi.com/article/10.3390/life15060930/s1, Figure S1. Characterization of synthesized IMPY (A) and SnMPY (B) using liquid chromatography-mass spectrometry (LC-MS). Figure S2. Characterization of synthesized IMPY using ^1^H-Nuclear Magnetic Resonance Spectroscopy (A and B) and ^13^C-Nuclear Magnetic Resonance Spectroscopy (C) where the expanded area of A in the range of δ 8.5–δ 6.0 is shown in (B). Figure S3. Characterization of synthesized SnMPY using ^1^H-Nuclear Magnetic Resonance Spectroscopy (A and B) and ^13^C-Nuclear Magnetic Resonance Spectroscopy (C) where the range of δ 3.5–δ 0 is shown in (A) and the range of δ 8.6–δ 6.4 is shown in (B).
