# Supplementary material for: Synthesis and Evaluation of 125I-IMPY: Application in Neuroblastoma Tracing and Treatment
Source: Life (Basel). 2025 Jun 9;15(6):930. doi: 10.3390/life15060930 (PMC12194121; doi:10.3390/life15060930)
Supplement: Supplementary file 1 [file life-15-00930-s001.zip › life-3589295-supplementary.pdf]

Supplementary figures

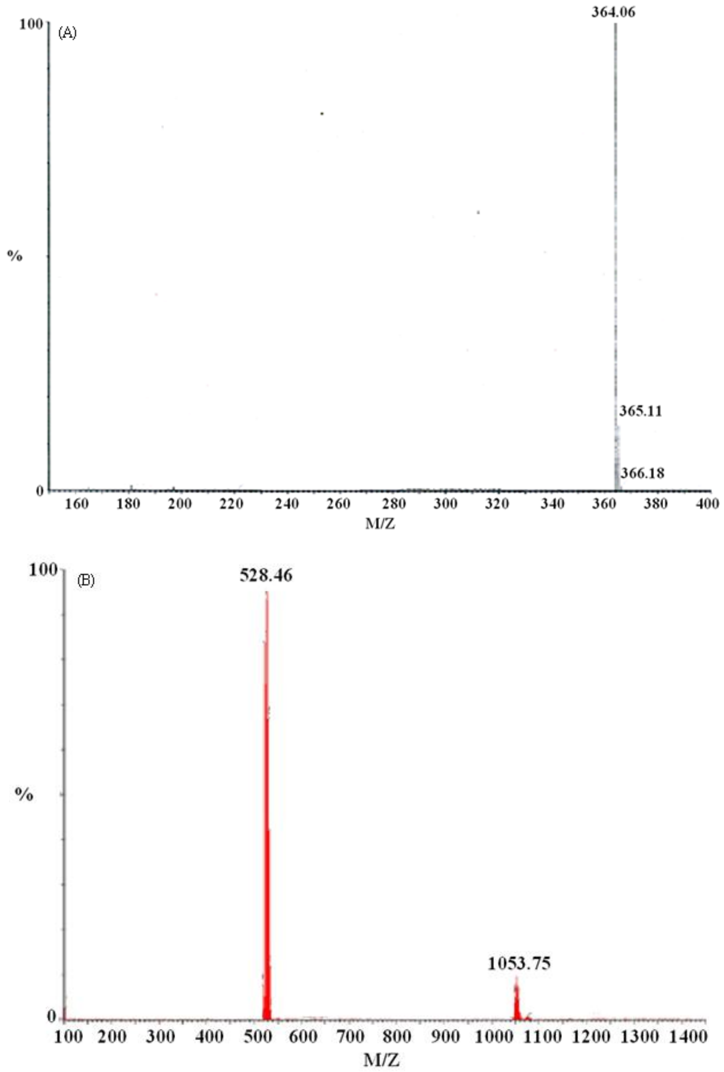

Supplementary Figure S1. Characterization of synthesized IMPY (A) and SnMPY (B) using liquid chromatography-mass spectrometry (LC-MS).

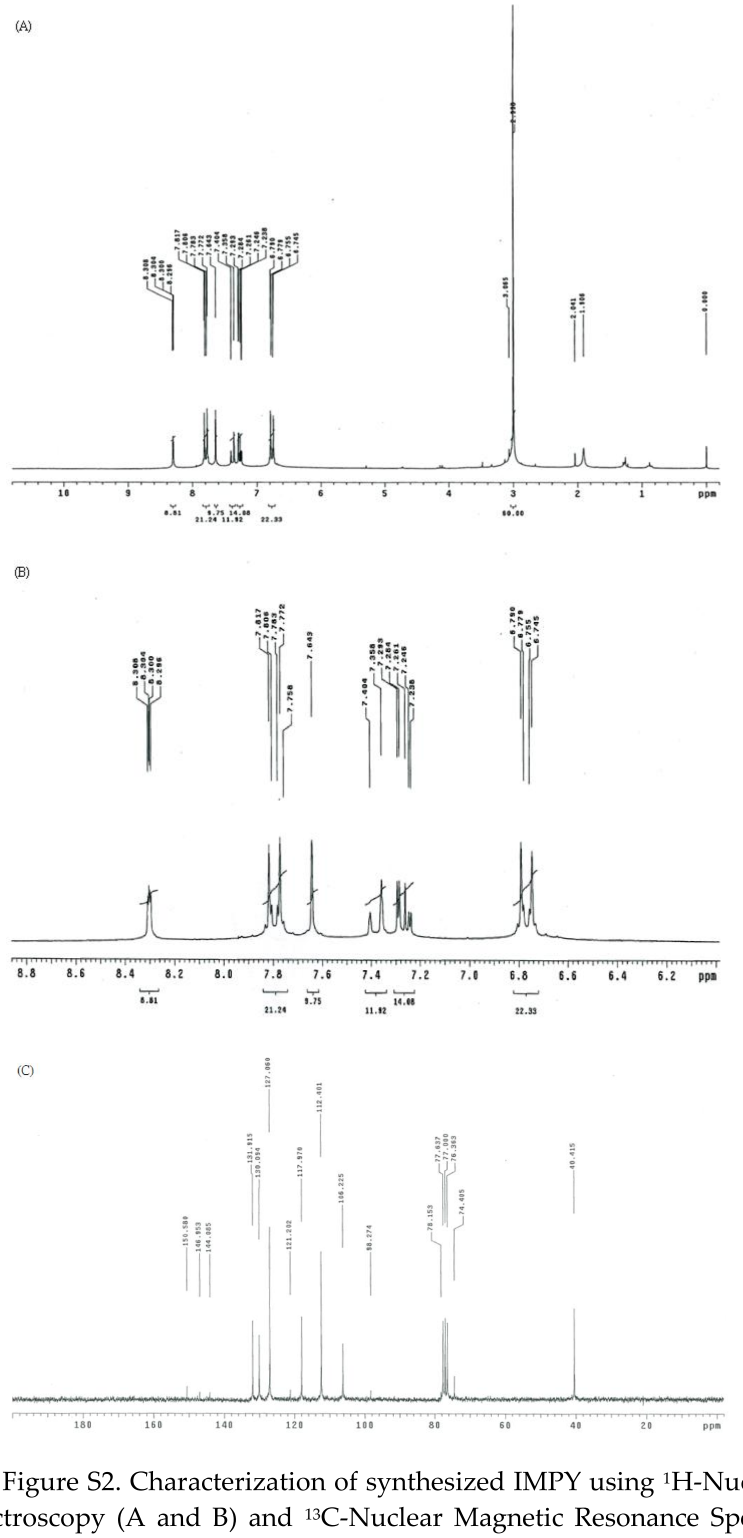

Supplementary Figure S2. Characterization of synthesized IMPY using ^{1}H-Nuclear Magnetic Resonance Spectroscopy (A and B) and ^{13}C-Nuclear Magnetic Resonance Spectroscopy (C) where the expanded area of A in the range of δ 8.5–δ 6.0 is shown in (B).

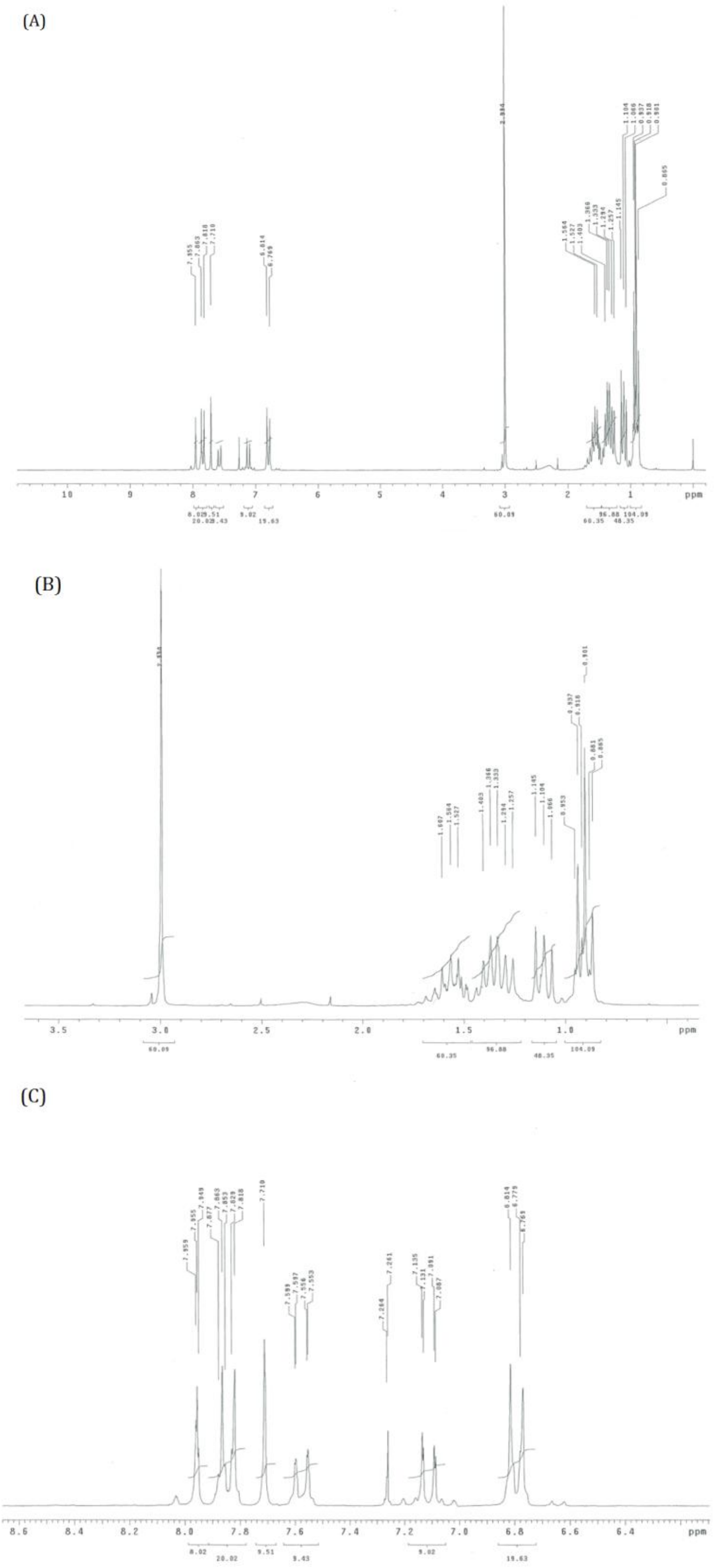

Supplementary Figure S3. Characterization of synthesized SnMPY using ^{1}H-Nuclear Magnetic Resonance Spectroscopy (A and B) and ^{13}C-Nuclear Magnetic Resonance Spectroscopy (C) where the range of δ 3.5–δ 0 is shown in (A) and the range of δ 8.6–δ 6.4 is shown in (B).
